# Soluble FAS ligand is not required for pancreatic islet inflammation or beta-cell destruction in non-obese diabetic mice

**DOI:** 10.1038/s41420-019-0217-z

**Published:** 2019-09-23

**Authors:** Prerak M. Trivedi, Stacey Fynch, Lucy M. Kennedy, Jonathan Chee, Balasubramanian Krishnamurthy, Lorraine A. O’Reilly, Andreas Strasser, Thomas W. H. Kay, Helen E. Thomas

**Affiliations:** 1 St. Vincent’s Institute, Fitzroy, Victoria 3065 Australia; 2 Department of Medicine, St. Vincent’s Hospital, The University of Melbourne, Fitzroy, Victoria 3065 Australia; 3 The Walter and Eliza Hall Institute of Medical Research, Parkville, Victoria 3050 Australia; 4 Department of Medical Biology, University of Melbourne, Melbourne, Australia; 5 Present Address: Memorial Sloan Kettering Cancer Center, New York, NY 10065 USA; 6 Present Address: University of Western Australia, Nedlands, Western Australia 6009 Australia

**Keywords:** Autoimmunity, Type 1 diabetes

## Abstract

CD8^+^ T cells play a central role in beta-cell destruction in type 1 diabetes. CD8^+^ T cells use two main effector pathways to kill target cells, perforin plus granzymes and FAS ligand (FASL). We and others have established that in non-obese diabetic (NOD) mice, perforin is the dominant effector molecule by which autoreactive CD8^+^ T cells kill beta cells. However, blocking FASL pharmacologically was shown to protect NOD mice from diabetes, indicating that FASL may have some role. FASL can engage with its receptor FAS on target cells either as membrane bound or soluble FASL. It has been shown that membrane-bound FASL is required to stimulate FAS-induced apoptosis in target cells, whereas excessive soluble FASL can induce NF-κB-dependent gene expression and inflammation. Because islet inflammation is a feature of autoimmune diabetes, we tested whether soluble FASL could be important in disease pathogenesis independent of its cell death function. We generated NOD mice deficient in soluble FASL, while maintaining expression of membrane-bound FASL due to a mutation in the FASL sequence required for cleavage by metalloproteinase. NOD mice lacking soluble FASL had normal numbers of lymphocytes in their spleen and thymus. Soluble FASL deficient NOD mice had similar islet inflammation as wild-type NOD mice and were not protected from diabetes. Our data indicate that soluble FASL is not required in development of autoimmune diabetes.

## Introduction

Type 1 diabetes results from destruction of insulin producing beta cells by autoreactive T cells^1,2^. Islet-specific CD8^+^ T cells are activated in the pancreatic draining lymph node and then migrate via the circulation to the pancreatic islets. Once in the islets, CD8^+^ T cells acquire effector function^3^ and directly interact with and kill beta cells by delivering cytotoxic molecules including perforin and granzymes^4–7^.

FasL is expressed on cytotoxic T cells and NK cells. Its expression on the cell surface is cleaved by metalloprotease to produce soluble FASL (sFASL)^8^. To study the roles of membrane-bound and soluble FASL independently, mouse models were generated where either form of FASL was absent^9^. Analysis of these animals showed that membrane-bound FASL is pro-apoptotic and is important for maintaining immune homoeostasis and killing target cells. In the non-obese diabetic (NOD) mouse model of autoimmune diabetes, blocking FAS signalling, and thus FAS-mediated apoptosis, in beta cells did not protect NOD mice from diabetes, indicating FAS-mediated beta-cell death is not important in diabetes pathogenesis^10–12^. The death receptor FAS can be upregulated on the surface of beta cells by pro-inflammatory cytokines, but it is not readily detectable in spontaneously diabetic NOD mice^12^. In contrast, perforin deficiency significantly protects from diabetes, which suggests it plays a dominant role in killing beta cells^4,5,7^.

However, pharmacological blockade of FASL was reported to protect NOD mice from diabetes^13^ suggesting that FASL signalling may have a role in autoimmune diabetes that is independent of beta-cell apoptosis. In mice lacking membrane-bound FASL, excess sFASL induced non-canonical signalling, triggered an NF-κB-dependent inflammatory response and exacerbated lupus-like autoimmunity^9,14,15^. Additional studies suggest that sFASL may increase calcium signalling and thus promote trafficking of immune or cancer cells in a death domain-independent way^16,17^. In contrast, sFASL inhibited the pro-inflammatory activity of mFASL in the tumour microenvironment^18^ and in retinal detachment, sFASL prevented mFASL-triggered photoreceptor cell death^19^. Also, a significant decrease in induction of diabetes was observed when diabetogenic splenocytes were treated ex vivo with sFASL^20^. To resolve its role in autoimmune diabetes, we generated NOD mice deficient in sFASL.

## Results

### Loss of soluble FASL does not affect immune homoeostasis in NOD mice

We generated NOD mice deficient in secreted FASL (sFASL) by backcrossing gene-targeted C57BL/6 mice unable to shed FASL from the surface of the cells while retaining cell surface membrane-bound FASL (mFASL) onto the NOD/Lt genetic background (NOD.*FasL*^∆*s/*∆*s*^). These mice have a mutation in the sequences required for metalloprotease-mediated cleavage in the *fasl* gene^9^.

**Fig. 1:**
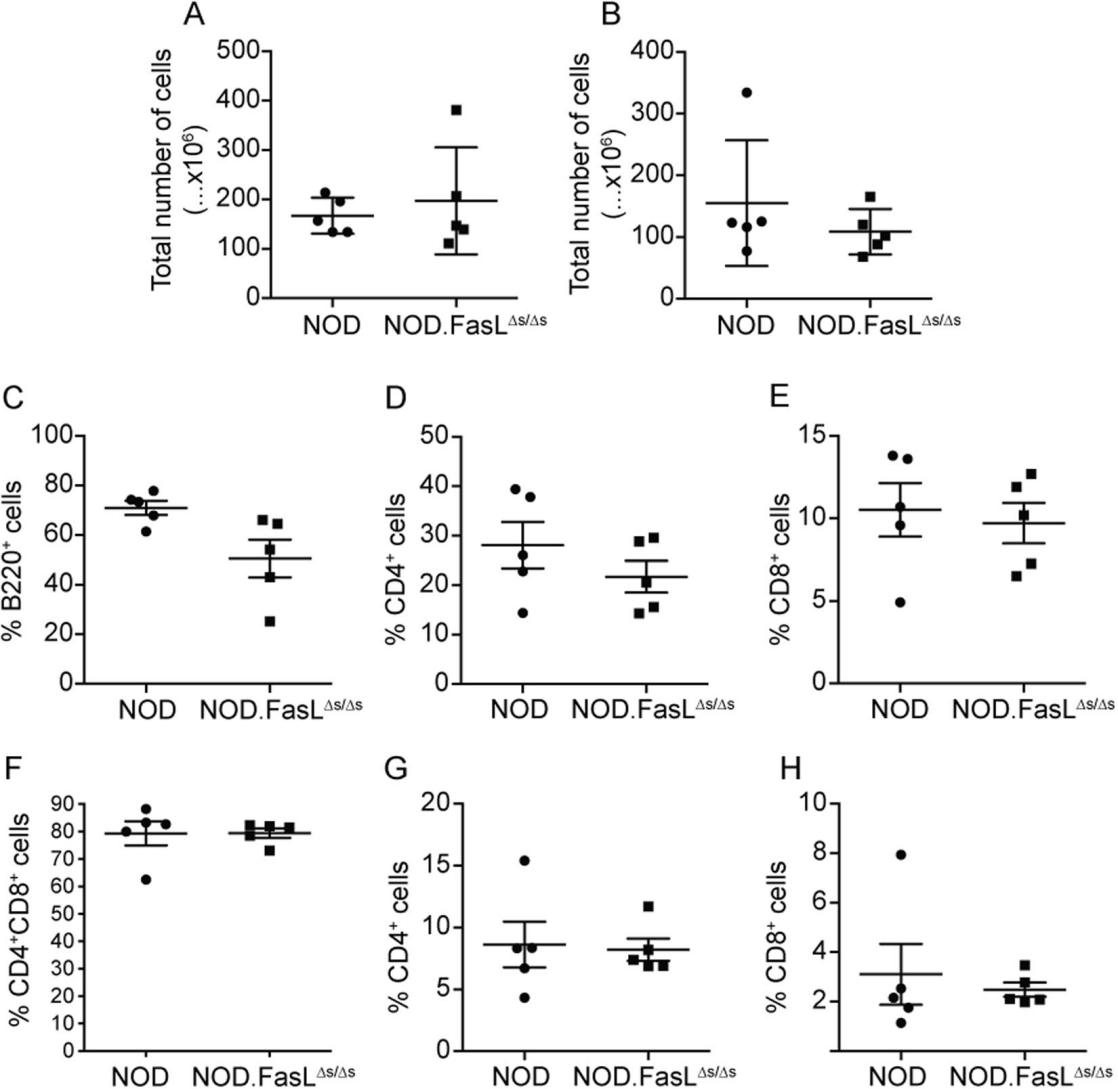
Normal immune homoeostasis in NOD.*FasL*^∆*s/*∆*s*^ mice. Spleen and thymus from 17–20-week-old NOD and NOD.*FasL*^∆*s/*∆*s*^ mice were used to study immune homoeostasis. **a**, **b** Total number of splenocytes (**a**) and thymocytes (**b**). **c** Percentage of B220^+^ cells in the spleen (*p* = 0.03, unpaired Student’s *t*-test). **d**, **e** Percentage of CD4^+^ and CD8^+^ T cells in the spleen. **f**–**h** Percentages of CD4^+^CD8^+^ double positive (**f**), CD4^+^ single positive (**g**) and CD8^+^ single positive (**h**) thymocytes. Data for individual mice are shown (*n* = 5 mice/group) with mean ± SEM, analysed by unpaired student’s *t*-test

Genetic deficiency of FASL overall, i.e. loss of both membrane-bound and soluble FASL (*FasL*^*gld/gld*^) has been shown to result in lymphadenopathy and loss of immune homoeostasis^9,13^. We confirmed that loss of sFasL did not affect immune homoeostasis in NOD. There was no change in the total number of cells in spleen (Fig. 1a) or thymus (Fig. 1b). NOD mice lacking functional FASL (*NOD.FasL*^*gld/gld*^) have accumulation of B220^+^TCR^+^ T cells in the spleen^13,21,22^. We did not observe an increase in these unconventional cells in NOD.*FasL*^∆*s/*∆*s*^ mice (not shown). The proportions of splenic B220^+^ B cells in NOD.*FasL*^∆*s/*∆*s*^ mice were significantly reduced compared to NOD mice (Fig. 1c). The proportions of CD4^+^ and CD8^+^ T cells in the spleen and thymus were not different to those found in wild-type NOD mice (Fig. 1d–h). Thus, loss of sFASL does not affect immune homoeostasis in NOD mice except for a minor reduction in the proportion of B220^+^ cells in the spleen.

### Loss of soluble FASL does not affect insulitis and diabetes in NOD mice

**Fig. 2:**
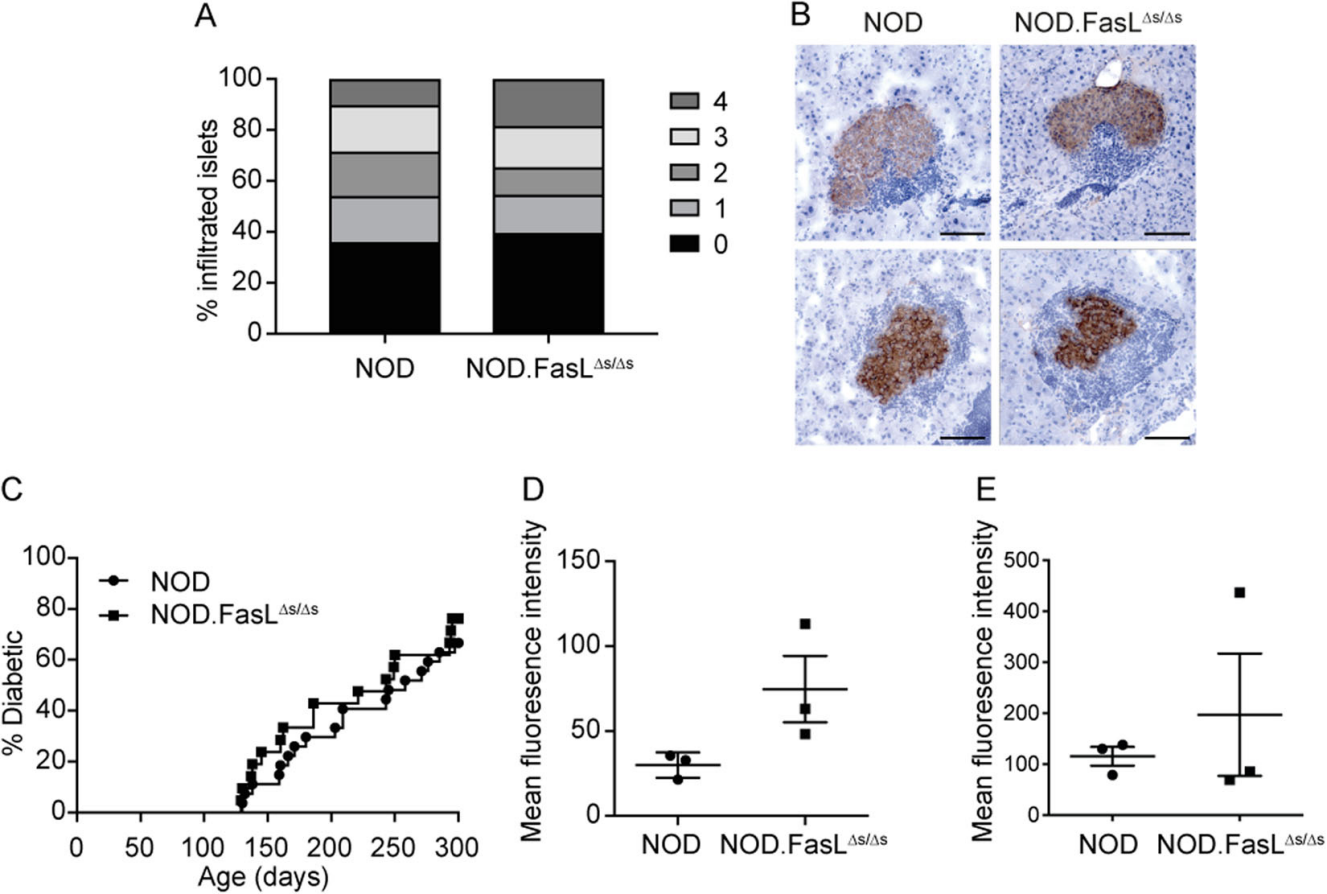
NOD.*FasL*^∆*s/*∆*s*^ mice are not protected from insulitis and diabetes. **a** Insulitis scores from 150-day-old female NOD and NOD.*FasL*^∆*s/*∆*s*^ mice. Data are not significantly different (two-way ANOVA). **b** Photographs of insulin-stained frozen sections showing insulitis in NOD (left panels) and NOD.*FasL*^∆*s/*∆*s*^ mice (right panels). Islets from two individual mice per genotype shown. Magnification 100 ×, scale bar 100 µm. **c** Cumulative spontaneous diabetes incidence in female NOD (*n* = 27) and NOD.*FasL*^∆*s/*∆*s*^ (*n* = 21) mice monitored until 300 days of age. Data are not significantly different (Log-rank Mantel-Cox test). **d**, **e** Expression of FasL (mean fluorescence intensity) on the surface of islet-infiltrating CD4^+^ (**d**) and CD8^+^ (**e**) T cells. Data not significantly different (unpaired Student’s *t*-test). Data for individual mice are shown with mean ± SEM

We next examined whether loss of sFASL affected islet inflammation as sFASL has been shown to induce activation of NF-κB^9,14^. There was no difference in islet inflammation (insulitis) in pancreas sections from 150-day-old wild-type NOD and NOD.*FasL*^∆*s/*∆*s*^ mice (Fig. 2a, b). Cumulative diabetes incidence was also not different between NOD.*FasL*^∆*s/*∆*s*^ and wild-type NOD mice (Fig. 2c). This is despite a slight but not significant increase in the expression of mFASL on the surface of islet-infiltrating CD4^+^ but not CD8^+^ T cells (Fig. 2d, e). This is consistent with a previous report that showed increased mFASL on activated T cells from FASL^∆*s/*∆*s*^ mice on a C57BL/6 background^9^. Our results suggest that sFASL does not play a significant role in islet inflammation or beta-cell destruction in the pathogenesis of autoimmune diabetes in NOD mice.

## Discussion

It has been suggested that sFASL can activate NF-κB and drive production of pro-inflammatory cytokines, and consistent with this, mice that lack mFASL and only express sFASL have high levels of active p65 NF-κB in their spleen and liver^9^. Because inflammation and pro-inflammatory cytokines are thought to drive autoimmunity, we studied the role of sFASL in autoimmune diabetes using NOD mice that lack sFASL while maintaining mFASL and normal immune homoeostasis. Our results indicate that sFASL does not play an important role in pathogenesis of autoimmune diabetes in NOD mice. Our data suggest that the potential activation of NF-κB by sFASL does not contribute markedly to islet inflammation in NOD mice. This is consistent with our previous findings suggesting that NF-κB activation in islets of spontaneously diabetic NOD mice is minimal^23^.

O’Reilly et al. showed that the expression of mFASL was increased on the surface of in vitro mitogen-activated T cells from mice that lack sFASL^9^, and we observed a similar increase in mFASL expression on islet-infiltrating CD4^+^ T cells. It is possible that this increased surface mFASL expression could result in increased ability of T cells to kill target cells and thus lead to accelerated diabetes. We saw small but significant decrease in the proportion of B220^+^ B cells in the spleen of mice lacking sFASL, which could be attributed to increased FASL-mediated death of B220^+^ cells. However, we did not observe any difference in diabetes incidence or insulitis in the NOD.*FasL*^∆*s/*∆*s*^ mice compared to wild-type NOD mice.

The FASL-FAS-induced cell death pathway is a key mechanism by which the number of immune cells is regulated^24^. NOD mice with mutation in FASL that abolishes the function of both mFASL and sFASL (NOD.*FasL*^*gld*/*gld*^) are protected from diabetes, but unconventional B220^+^CD4^−^CD8^−^TCR^+^ T cells accumulate in these mice^25^ and the disturbed immune homoeostasis most likely accounts for protection from diabetes. However, it was also shown that blocking FASL with a neutralising antibody could protect NOD mice from diabetes without loss of immune homoeostasis^13,26^. Hamad and colleagues concluded that anti-FASL Ab treatment increased the number of IL-10 secreting CD5^+^ B cells in the pancreas. However, inhibiting IL-10 receptor signalling in anti-FASL Ab treated mice did not induce diabetes^26^, so it is still unclear how anti-FASL antibody induces its therapeutic effect in NOD mice. Our results suggest that the therapeutic effect of anti-FASL antibody must be achieved by inhibiting mFASL, because genetic loss of sFASL only did not prevent diabetes development.

Previous in vitro studies have shown that sFASL can keep FAS^+^CD4^+^CD45RB^low^ memory cells in check by inducing their death^20^. If this were the case, loss of sFASL might have resulted in accelerated diabetes in NOD mice, which was not the case in our study. Thus, we conclude that sFASL is unlikely to be involved in homoeostasis of islet autoantigen-specific memory T cells in vivo. In conclusion, our results suggest that while deficiency of sFASL does not alter immune cell homoeostasis, it also does not have a major effect on the pathogenesis of autoimmune diabetes in NOD mice.

## Materials and methods

### Mice and diabetes monitoring

All animal studies were conducted at St. Vincent’s Institute and approved by the institutional animal ethics committee. Soluble FASL deficient C57BL/6 mice have been described previously^9^ and were backcrossed onto the NOD/Lt genetic background for >10 generations to generate sFASL-deficient NOD mice (NOD.*FasL*^∆*s/*∆*s*^). DNA from backcrossed mice was genotyped using the Illumina mouse medium density linkage panel for single nucleotide polymorphisms and strain differences were identified using The Jackson Laboratory Mouse Genome Informatics and National Center for Biotechnology Information databases (NCBI37/mm9 assembly). Backcrossed mice were of the NOD/Lt genotype across the whole genome except for the region on chromosome 1 between and including ~136.1 (rs13476119) and 197.1 Mb (mCV24145570) encompassing the *Fasl* locus.

Female mice were monitored for spontaneous diabetes by urine glucose measurement using Diastix (Bayer Diagnostics, UK). Diabetes was confirmed by two consecutive blood glucose readings higher than 15 mmol/L, using Advantage II Glucose Strips (Roche, Basel, Switzerland).

### Flow cytometry

Single cell suspensions of spleen or thymus were prepared and stained with antibodies using standard procedures. Antibodies used were anti-CD4 (RM4-5), anti-CD8a (5H10) and anti-B220 (RA3-6B2) (all from BD biosciences San Jose, CA). FASL on islet-infiltrating T cells was detected using anti-CD178.1 (Kay-10, Biolegend). Flow cytometry was performed on a BD Fortessa cell analyser and data were analysed using FlowJo software (TreeStar).

### Histology and insulitis scoring

Pancreata from 150-day-old female mice were collected and frozen in optimal cutting temperature (OCT) compound (Tissue-Tek®, Sakura). Five-micrometer cryostat sections were cut at three levels, 200 μm apart. Sections were stained with guinea pig anti-insulin Ab (Dako Cytomation, CA) followed by horse radish peroxidase-anti-guinea pig Ig (Dako Cytomation). Insulitis scoring was performed in a blinded manner based on the following criteria: score 0, no infiltrate; score 1, peri-islet infiltrate; score 2, extensive peri-islet infiltrate; score 3, intra-islet infiltrate and score 4, extensive intra-islet infiltrate or total beta-cell loss. Sections were photographed using a Leica camera at ×100 magnification.

### Statistical analysis

Data were analysed using GraphPad Prism software (GraphPad Software Inc., San Diego, CA). Values are given as mean ± SEM. Groups were compared using Student’s *t*-test or one-way ANOVA where appropriate. Diabetes development was analysed by the log-rank test.
